# Induced Sputum Multi-Omics Reveals Airway Signatures of COPD in Smokers: A Pilot Study

**DOI:** 10.3390/ijms27052271

**Published:** 2026-02-28

**Authors:** Kaja Pulik, Piotr Korczyński, Katarzyna Mycroft-Rzeszotarska, Iga Ciesielska-Markowska, Magdalena Kucia, Magdalena Paplińska-Goryca, Diana Wierzbicka, Kannathasan Thetchinamoorthy, Zofia Wicik, Katarzyna Górska

**Affiliations:** 1 Department of Internal Medicine, Pulmonary Diseases and Allergy, Medical University of Warsaw, Banacha 1A, 02-097 Warsaw, Poland; katarzyna.mycroft@gmail.com (K.M.-R.); magdalena.paplinska@wum.edu.pl (M.P.-G.); 2 Department of Pulmonary Diseases, Internal Medicine, Thoracic Oncology and Transplantology, National Medical Institute of the Ministry of Interior and Administration (MSWiA), Wołoska 137, 02-507 Warsaw, Poland; piotr.korczynski@pimmswia.gov.pl (P.K.); drkpgorska@gmail.com (K.G.); 3 Laboratory of Regenerative Medicine, Medical University of Warsaw, Banacha 1B, 02-097 Warsaw, Poland; magdalena.kucia@wum.edu.pl (M.K.); diana.wierzbicka@wum.edu.pl (D.W.); kannathasan.thetchinamoorthy@wum.edu.pl (K.T.); 4 Department of Experimental and Clinical Pharmacology, Center for Preclinical Research and Technology CEPT, Medical University of Warsaw, 02-097 Warsaw, Poland; zwicik@ipin.edu.pl; 5 Department of Experimental and Clinical Neuroscience, Institute of Psychiatry and Neurology, Sobieskiego 9 Street, 02-957 Warsaw, Poland

**Keywords:** COPD, multi-omics, induced sputum, proteomics, metabolomics, lipidomics, pathway enrichment

## Abstract

Chronic obstructive pulmonary disease (COPD) is a leading cause of mortality worldwide, yet only a fraction of smokers develops the disease, suggesting protective mechanisms in resilient individuals. Identifying airway-localized molecular signatures may improve our understanding of disease pathomechanisms and support hypothesis generation for biomarker research. In this pilot study, induced sputum from smokers with COPD (*n* = 28) and smokers without COPD (*n* = 16; Global Initiative for Chronic Obstructive Lung Disease (GOLD)-defined pre-COPD) was analyzed by untargeted proteomics, metabolomics, and lipidomics. After quality control, 1180 proteins, 187 metabolites, and 1234 lipids were retained. Analyses included univariate models with false discovery rate adjustment and multivariate analyses (PCA, PLS-DA), followed by pathway enrichment and protein interaction network analysis. While few features remained significant after FDR correction, consistent cross-omics patterns were observed. COPD was characterized by ↑ glutathione, creatine, and L-arginine; ↓ CCDC88A and ↑ STAT3 and SYDE2; and broad lipid remodeling involving phosphatidylcholines, sphingolipids, and eicosanoids. Network analysis highlighted STAT3 as a highly connected node linking COPD-related genes. These findings suggest that the multi-omic profiling of induced sputum can capture coherent airway-localized molecular signatures such as oxidative stress, cytoskeletal remodeling, and Rho-family GTPase signaling. However, the results should be interpreted as exploratory and require validation in functional studies.

## 1. Introduction

Chronic obstructive pulmonary disease (COPD) is a major global cause of mortality, responsible for approximately 3.5 million deaths annually [1,2]. Although heavy smoking is the predominant risk factor, only a subset of smokers develops COPD, implying the existence of molecular defense mechanisms that may prevent disease onset [3]. Current diagnostic tools, such as post-bronchodilator spirometry, fail to detect early stages of disease and lack objective biomarkers, thereby delaying recognition and treatment [4,5]. Despite therapeutic advances, no intervention halts or reverses structural changes [6].

At the molecular level, chronic oxidative stress is associated with widespread disturbances in lipid, protein, and small-molecule metabolism within the airway epithelium [7,8,9]. Experimental and human studies show that oxidative stress promotes ceramide generation through the activation of sphingomyelinases, mitochondrial dysfunction, and redox-related alterations in amino-acid and energy pathways [7,10]. These processes coincide with shifts in extracellular-matrix turnover and cytoskeletal organization, which are frequently observed in COPD and relate to airflow obstruction and emphysema [11,12,13]. Although these pathways are not yet fully linked by a single mechanistic cascade, the current evidence suggests that chronic smoke exposure affects multiple interconnected molecular networks that impair epithelial resilience, repair capacity, and tissue maintenance [14,15].

Multi-omics profiling offers opportunities to detect molecular processes associated with diseases; however, most studies focus on single molecular layers and blood samples, thereby overlooking airway-localized processes. Induced sputum provides a minimally invasive, lung-specific matrix, yet integrated proteomic, metabolomic, and lipidomic analyses in this material remain limited [16].

In this pilot study, we applied an integrative multi-omics approach combining the proteomic, metabolomic, and lipidomic profiling of induced sputum from smokers with and without COPD. Using univariate and multivariate analyses followed by pathway enrichment and protein interaction network analysis, we aimed to explore airway-localized molecular alterations differentiating COPD from pre-COPD smokers and identify cross-omics molecular patterns that may be associated with COPD-related airway remodeling.

## 2. Results

### 2.1. Clinical Characteristics

A total of 44 participants with smoking history were included (COPD *n* = 28; pre-COPD *n* = 16). Baseline demographics and comorbidities are shown in Table 1. Groups were comparable with respect to age, sex, and BMI, with no statistically significant differences observed. COPD participants had a greater cumulative smoking history (*p* = 0.035), higher mMRC dyspnea scores (*p* < 0.001), and higher CAT scores (*p* = 0.007).

### 2.2. Pulmonary Function and Imaging

Pulmonary function and CT imaging results are presented in Table 2. COPD patients exhibited significantly lower FEV_1_, reduced FEV_1_/FVC ratio, higher residual volume (RV), and greater emphysema percentage values compared with pre-COPD smokers.

### 2.3. Metabolomics

#### 2.3.1. Univariate Analysis

A total of 492 annotated metabolites were included in the analysis. Data preprocessing involved the removal of features with excessive missing values (>50% per group), imputation of missing values using 20% of the minimum positive value, and normalization to the total ion signal per sample. After quality control and filtering, 187 metabolites remained for downstream statistical analysis.

Univariate comparison identified several metabolites with nominal significance (*p* < 0.05): decreased in COPD—PC 15:0/22:6 (*p* = 0.008), N-(13Z-docosanoyl)-ethanolamine (*p* = 0.032); increased in COPD—glutathione (*p* = 0.021), isobutyryl-L-carnitine (*p* = 0.036), and creatine (*p* = 0.038) (see Supplementary Table S1). However, none of these metabolites remained significant after correction for multiple testing (all FDRs = 0.70).

**Figure 1:**
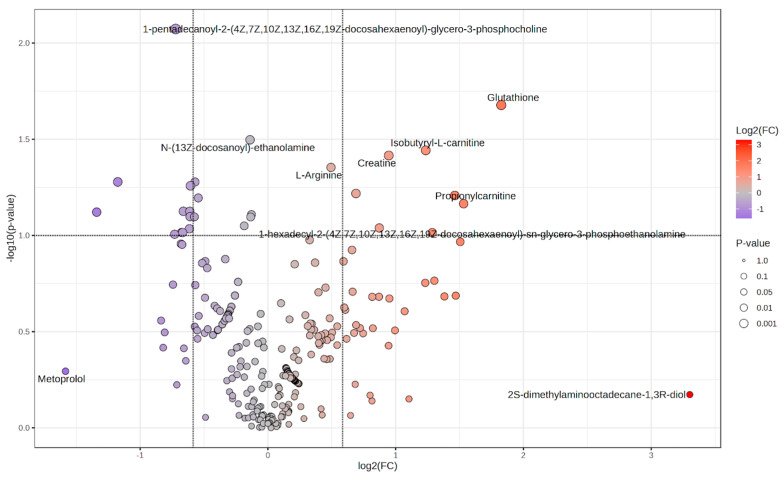
Volcano plot of differential metabolite levels between COPD and pre-COPD patients (log_2_ fold change vs. −log_10_ *p*-value).

**Figure 2:**
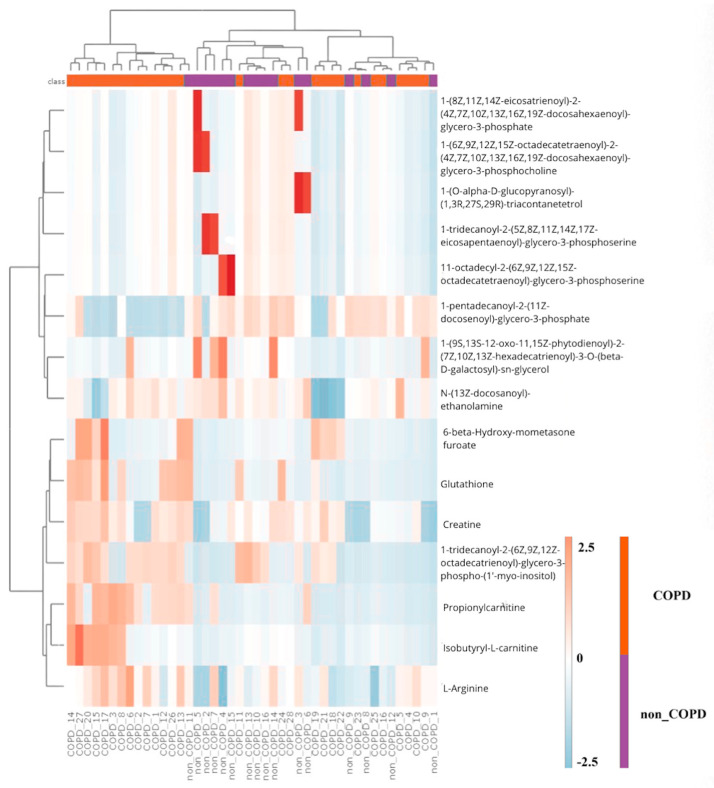
Hierarchical clustering heatmap between COPD and pre-COPD groups based on metabolomic analysis of induced sputum. The rows and columns are ordered based on the results of hierarchical clustering with dendrograms for the patients shown on the horizontal axis and laboratory data shown on the vertical axis. The color scale codes the value of a variable with blue corresponding to the lowest value.

The volcano plot displays the distribution of log_2_ fold changes and statistical significance (–log_10_ *p*-values) across all annotated metabolites, comparing COPD and pre-COPD subjects (Figure 1). A hierarchical clustering heatmap based on the top nominal metabolites shows partial grouping by clinical status with considerable overlap (Figure 2).

#### 2.3.2. Multivariate Analysis

Principal component analysis (PCA) did not reveal clear separation between groups, with a broad overlap of 95% confidence ellipses (Supplementary Figure S1; PC1 = 20.3%, PC2 = 9.1%). In contrast, partial least squares–discriminant analysis (PLS-DA) suggested a trend toward separation between COPD and pre-COPD groups, with non-overlapping confidence ellipses (Supplementary Figure S2; Component 1 = 5.0%, Component 2 = 16.3%). These multivariate patterns should be interpreted cautiously as they were observed in the absence of FDR-significant individual features.

### 2.4. Lipidomic Analysis

#### 2.4.1. Univariate Analysis

A total of 1248 annotated lipids were included in the multi-omics analysis. Data preprocessing involved the removal of features with excessive missing values (>50% per group), imputation of missing values using 20% of the minimum positive value, and normalization to the total ion signal per sample. After quality control and filtering, 1234 lipids remained for downstream statistical analysis.

**Figure 3:**
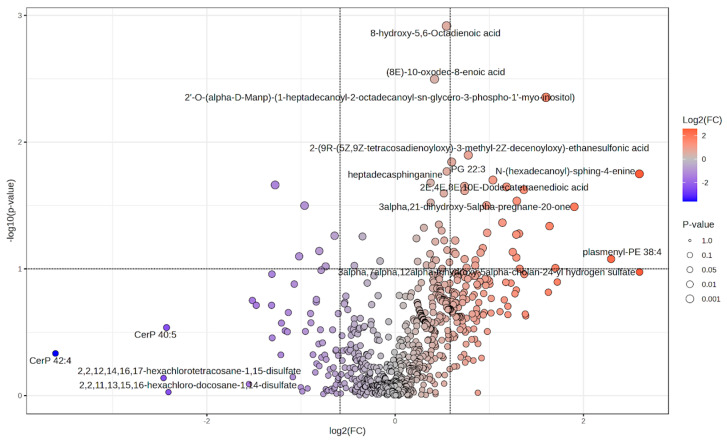
Volcano plot of differential lipid abundance between COPD and pre-COPD subjects. Points represent individual lipid species; color indicates direction and magnitude of log_2_ fold change; size reflects *p*-value.

Univariate tests identified several lipid species with nominal significance (*p* < 0.05). Selected sphingolipids were higher in the COPD group whereas certain ceramide-1-phosphate species (e.g., CerP 42:4, CerP 40:5) were lower. A volcano plot for all annotated lipids is shown in Figure 3; top features are listed in Supplementary Table S2. Although several species showed large fold changes and low *p*-values, none remained significant after FDR correction.

**Figure 4:**
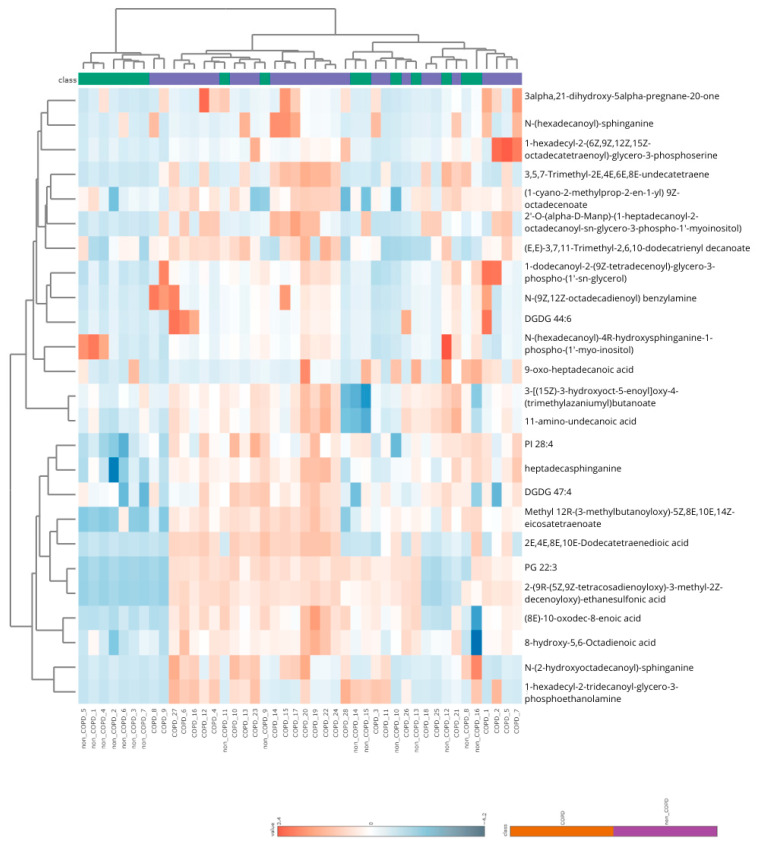
Hierarchical clustering heatmap between COPD and pre-COPD groups based on lipidomic analysis of induced sputum. The rows and columns are ordered based on the results of hierarchical clustering with dendrograms for the patients shown on the horizontal axis and laboratory data shown on the vertical axis. The color scale codes the value of a variable with blue corresponding to the lowest value.

A hierarchical clustering heatmap (Figure 4) revealed partial group separation. COPD samples tended to cluster with a higher abundance of sphingolipids and unsaturated fatty acids whereas pre-COPD samples showed relative enrichment in selected ceramide classes.

#### 2.4.2. Multivariate Analysis

The principal component analysis (PCA) of the lipidomic dataset did not reveal distinct group separation, with substantial overlap between COPD and pre-COPD confidence ellipses (Supplementary Figure S3; PC1 = 30.8%, PC2 = 6.7%). Partial least squares–discriminant analysis (PLS-DA) suggested partial separation, with COPD and pre-COPD samples tending toward different regions of the scores plot, though the 95% confidence ellipses still overlapped (Supplementary Figure S4; Component 1 = 27.6%, Component 2 = 5.9%). These multivariate patterns were observed in the absence of FDR-significant univariate features.

### 2.5. Proteomic Profiling of Induced Sputum

#### 2.5.1. Protein Identification, Normalization, and Filtering

In the initial dataset, 15,400 protein features were detected across all sputum samples. After quality filtering, including the removal of low-confidence identifications and proteins with >50% missing values per group, 1180 annotated proteins remained. Intensities were normalized to total protein content, measured independently by BCA assay.

#### 2.5.2. Univariate Analysis

**Figure 5:**
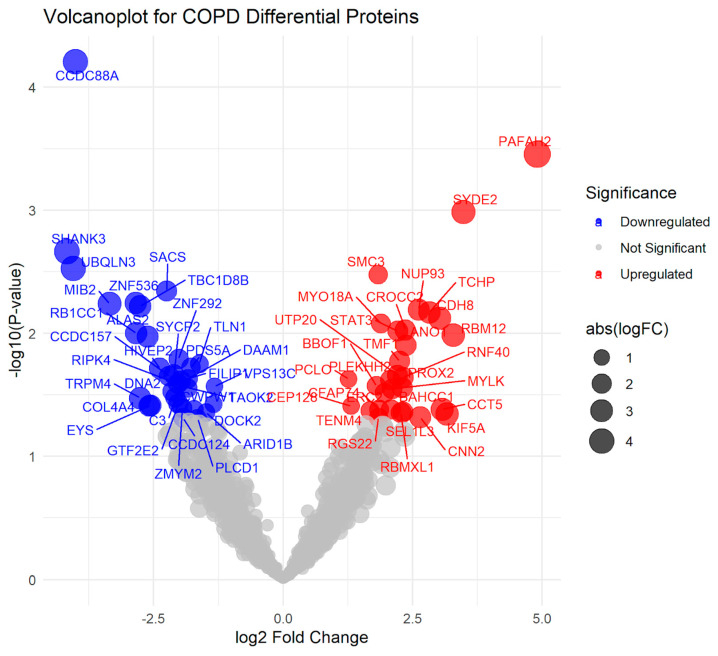
Volcano plots of differentially expressed proteins between COPD and pre-COPD patients. The x-axis shows log_2_ fold change and the y-axis shows −log_10_ *p*-values. Point size represents the absolute value of log_2_ fold change (abs (logFC)), indicating how large the expression difference is, regardless of direction. Colors indicate upregulated and downregulated proteins.

**Figure 6:**
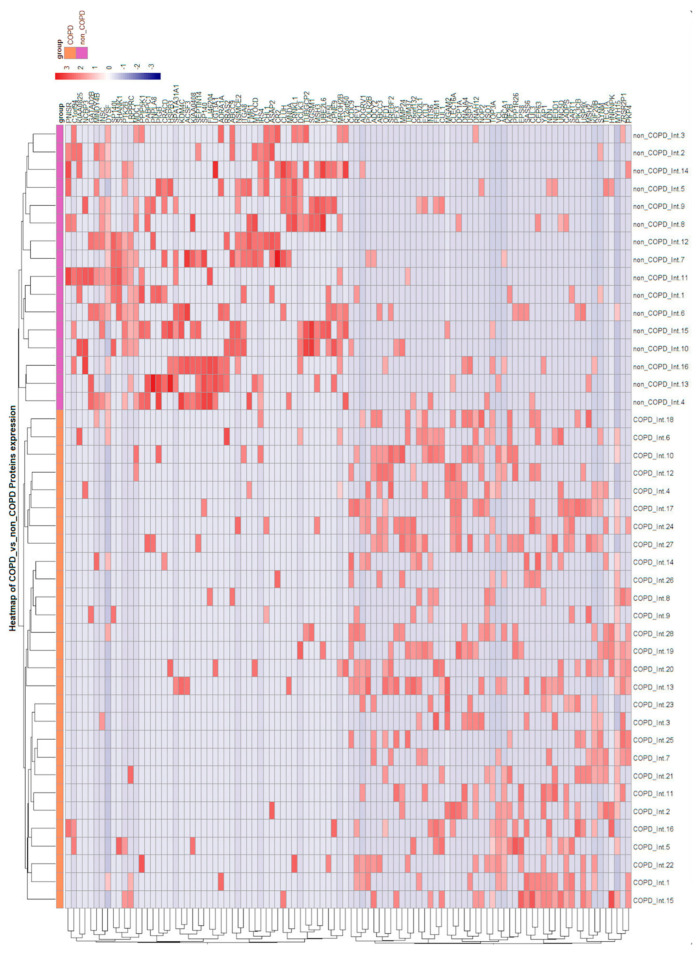
Heatmap of top 70 proteins ranked by *p*-value and log_2_FC.

Group comparisons (unpaired two-tailed t-tests) identified 62 proteins at nominal significance *p* < 0.05 and log_2_FC > 1:32 proteins were decreased in COPD (CCDC88A (log_2_FC = −4.01, *p* = 0.0001), SHANK3, UBQLN3, ALAS2, and MIB2) and 30 proteins were increased (PAFAH2 (log_2_FC = 4.91, *p* = 0.0004), SYDE2, SMC3, STAT3, and CDH8). None remained significant after FDR correction, consistent with the modest sample size. See Figure 5 (volcano) and Figure 6 (heatmap of top 70 by *p*-value and effect size).

#### 2.5.3. Pathway Enrichment Analysis

**Figure 7:**
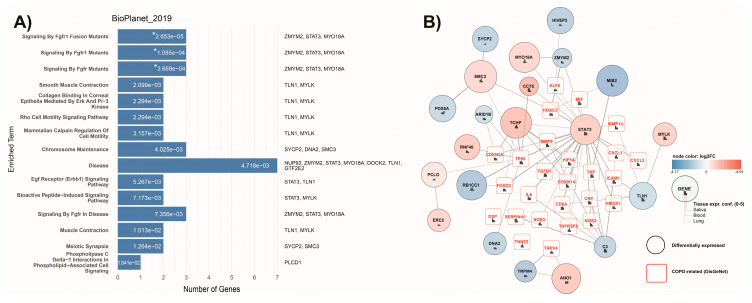
Pathway enrichment analysis and interaction network of differentially expressed proteins. (**A**) Pathway enrichment analysis results for the top 15 most significant signaling pathways. On the right side, genes associated with differentially expressed proteins are listed. Pathways marked with an asterisk were significant after adjusted correction. In panel (**A**), the left column indicates BioPlanet pathway category labels (e.g., ‘disease’ denotes disease-associated pathway class, not COPD specifically). (**B**) Interaction network between differentially expressed proteins (round shape) and COPD-related genes obtained from the DisGeNet database (square shape). The size of the node is associated with the *p*-value (larger values indicate lower *p*-values). Color of the nodes is associated with log_2_ fold change.

Pathway enrichment analysis highlighted pathways related to FGFR-associated signaling, smooth muscle contraction, cytoskeletal organization, and Rho GTPase-mediated cell motility (Figure 7A).

#### 2.5.4. Interaction Network Analysis

Interaction network analysis was performed to evaluate interactions between differentially expressed proteins’ related genes and COPD-related genes obtained from DisGeNet database. The top interactor based on connectivity was STAT3, showing interactions with twenty-three other nodes including four differentially expressed genes (TCHP, CCT5, MTO18A, ZMYM2), C3 showing interactions with eight nodes, and TCHP showing interactions with seven nodes (Figure 7B).

## 3. Discussion

In this pilot study, we used an integrative multi-omics approach on induced sputum from smokers with COPD and from symptomatic smokers without airflow limitation (pre-COPD), to explore airway-localized molecular differences between these groups. In this study, COPD was defined by post-bronchodilator FEV_1_/FVC levels below the lower limit of normal whereas pre-COPD referred to symptomatic smokers without airflow obstruction, as defined in the Materials and Methods section. Although few individual features remained significant after multiple testing correction, the integrative analysis of proteomic, metabolomic, and lipidomic data indicated biologically coherent patterns across omics layers. These observations support induced sputum as a valid and informative matrix for the molecular profiling of the airway disease [9,17].

The recurring cross-omic signals differentiating COPD from pre-COPD involved cytoskeletal regulation and membrane lipid remodeling. Proteomic analyses showed decreased levels of structural and polarity-associated proteins including CCDC88A and SHANK3, together with alterations in Rho-family GTPase regulators pointing to differences in cytoskeletal control. Consistent with these observations, pathway enrichment analyses indicated FGFR-dependent signaling, epithelial Erk and PI3K pathways, and Rho-mediated cell motility—processes essential for actin organization, cell adhesion, and epithelial stability [12,18,19]. Lipidomic profiling provided a complementary perspective on this axis. COPD samples exhibited a higher abundance of selected sphingolipids, including sphingomyelins, alongside reduced ceramide-1-phosphate species, consistent with the dysregulation of sphingolipid metabolism [8,20]. In contrast, pre-COPD samples showed a relative enrichment of selected ceramide classes in hierarchical clustering, highlighting heterogeneity within the sphingolipid axis. Downstream intermediates can exert opposing biological effects—ceramides are generally pro-inflammatory and pro-apoptotic, whereas S1P promotes cell survival and repair [7,20]. Collectively, these observations suggest that structural alterations in COPD may reflect coordinated disturbances in membrane composition and cytoskeletal signaling rather than isolated molecular changes.

Interaction network analysis identified STAT3 as a central hub linking proteins differentially expressed between COPD and pre-COPD cases with genes previously associated with COPD. STAT3 integrates inflammatory, stress-related, and growth-factor-mediated signaling, thereby connecting membrane-associated signaling and cytoskeleton-regulating processes with downstream transcriptional responses. In linear proteomic analyses, STAT3 showed a trend towards increased expression in COPD, consistent with the sustained activation of the JAK/STAT axis reported in chronic pulmonary diseases [11,21]. Taken together, these network-level findings support the hypothesis that structural remodeling, lipid dysregulation, and inflammatory signaling are may be functionally linked within airway tissue.

Changes related to oxidative and redox balance were also observed. Metabolomic analyses demonstrated increased levels of glutathione, creatine, and L-arginine in COPD, which may reflect increased antioxidant demand, metabolic adaptation, and the modulation of nitric-oxide-related processes. These metabolic differences were observed alongside proteomic signals involving redox sensitive signaling components. Together, these findings suggest coordinated metabolic and signaling responses within the airways, consistent with the established role of oxidative stress in COPD pathophysiology [7,22,23].

Strengths of this study included its integrative design spanning proteomics, metabolomics, and lipidomics within a single, clinically accessible airway matrix. Induced sputum is a minimally invasive biospecimen that directly reflects local airway biology. The deliberate selection of a pre-COPD comparison group, rather than a general population of smokers, reduces smoking-related background effects and improves the ability to explore airway-localized molecular differences related to disease presence and susceptibility. Limitations include the exploratory nature of this study and the relatively small cohort size inherent to its pilot design, requiring interpretation based on nominal *p*-values supported by biological consistency. The heterogeneous cellular composition of induced sputum was not explicitly addressed, and external validation was not performed. These limitations highlight the need for larger, longitudinal studies integrating cellular, molecular, and clinical data to validate and extend the present findings.

In conclusion, this pilot study showed that the multi-omics analysis of induced sputum can capture biologically coherent molecular patterns differentiating COPD from pre-COPD. Integrating proteomic, metabolomic, and lipidomic data revealed coordinated alterations in cytoskeletal regulation, membrane–lipid remodeling, and oxidative stress pathways. These findings support the use of induced sputum as an informative matrix for airway-focused molecular profiling and provide a rationale for validation in larger cohorts.

## 4. Materials and Methods

### 4.1. Study Design, Population and Definitions

This prospective observational study was part of a larger cohort investigation. Participants were recruited from the outpatient clinic at the Department of Internal Medicine, Pulmonary Diseases, and Allergy of the Medical University of Warsaw between February 2022 and March 2023. The study compared smokers with COPD to the pre-COPD group. Inclusion criteria (applied to both groups) were age ≥ 40 years, smoking exposure ≥ 10 pack-years, presence of respiratory symptoms (e.g., exertional dyspnea or chronic cough), and written informed consent. Pack-years were calculated as the number of cigarette packs smoked per day multiplied by the number of years of smoking, with one pack defined as 20 cigarettes.

COPD was diagnosed according to the ERS/ATS 2021 and Polish Respiratory Society 2012 guidelines as post-bronchodilator FEV_1_/FVC below the lower limit of normal (LLN) [24,25]. Pre-COPD was defined as post-bronchodilator FEV_1_/FVC ≥ LLN together with ≥ 1 of the following: modified Medical Research Council (mMRC) ≥ 1 or COPD Assessment Test (CAT) ≥ 10 or/and high-resolution computed tomography (HRCT) abnormalities consistent with early COPD (e.g., emphysema or airway wall thickening) or/and physiological abnormalities, i.e., DLCO z-score < −1.645 and/or evidence of hyperinflation [26,27]. Patients with COPD were further classified into GOLD 2022 ABE groups [28].

Exclusion criteria were a history of asthma or other chronic lung diseases, oral steroid use within three months, respiratory infection or COPD exacerbation within three months, connective tissue diseases, malignancy within five years, uncontrolled cardiovascular disease, chronic rhinosinusitis, contraindications to sputum induction, and alpha-1 antitrypsin deficiency. Exclusion criteria were verified based on patient history, medical records, and relevant tests.

The study protocol was approved by the Institutional Review Board (KB/124/2020). Written informed consent was obtained from all participants. The study was registered on ClinicalTrials.gov (identifier: NCT06826560) before recruitment commenced.

### 4.2. Induced Sputum and Blood Collection

All participants were advised to withhold inhaled medications on the morning of the procedure. Sputum induction was performed according to standardized methodology using stepwise inhalation of hypertonic saline (3%, 4%, 5%) via an ultrasonic nebulizer (Tajfun 1 MU 1, PPU MEDBRYT, Warsaw, Poland), following pre-treatment with 0.4 mg salbutamol [29,30]. Samples were processed using standardized protocols and stored at −80 °C (proteome and metabolome) or −20 °C (lipidome) until analysis. Detailed protocols, including sample processing conditions, solvent compositions, and instrument settings, are provided in Supplementary Methods M1–M3.

### 4.3. Lipidomic Analysis

Lipid extraction followed the methyl tert-butyl ether method with modifications described in Supplementary Method M1 [31]. Mass spectra were acquired in both positive and negative ESI modes on a 12 T SolariX Fourier transform ion cyclotron resonance (FTICR) mass spectrometer (Bruker Daltonics, Billerica, MA, USA). Lipid annotation was performed using the Lipid Maps 2018 and Bruker Lipid MetaboScape databases with false-positive removal in MetaboScape 5.0 (Bruker Daltonics).

### 4.4. Metabolomic Analysis

Sample preparation was performed using ice-cold methanol precipitation as described in Supplementary Method M2 [32]. Untargeted metabolomics was conducted on the same 12 T SolariX FTICR MS platform with dual-polarity electrospray ionization (ESI) acquisition. Annotation used the HMDB and Lipid Maps databases. Quality control included calibration before each run and analysis of pooled QC samples.

### 4.5. Proteomic Analysis

Protein precipitation, reduction/alkylation, and tryptic digestion followed established protocols, detailed in Supplementary Method M3 [33]. Peptides were analyzed by nano-ultra-high-performance liquid chromatography (UHPLC) (nanoElute, Bruker Daltonics) coupled via CaptiveSpray to an ESI-quadrupole time-of-flight (ESI-QTOF) mass spectrometer (Compact, Bruker Daltonics). Protein identification was performed in ProteinScape using Mascot (Matrix Science, London, UK) against the Homo sapiens SwissProt database.

### 4.6. Blood Collection and Biochemical Assays

For group characterization, venous blood samples were collected for standard laboratory assessments, including complete blood count, metabolic panel, lipid profile, NT-proBNP, CRP, albumin, and total protein. These laboratory assessments were obtained from all participants on the day of sputum induction.

### 4.7. Statistical Analysis

#### 4.7.1. Metabolomics and Lipidomics

Untargeted metabolomic and lipidomic profiling of induced sputum samples was performed. For both datasets, features present in <50% of samples in either group were removed prior to analysis. Remaining missing values were imputed using the 20% limit of detection (LoD) approach [34,35]. Data were normalized by total ion current, log_2_-transformed, and Pareto-scaled prior to statistical analysis.

#### 4.7.2. Proteomics

Raw liquid chromatography–tandem mass spectrometry (LC–MS/MS) data were processed to identify and annotate peptide features. Proteins detected in <50% of samples per group were excluded. Missing values were imputed using 20% of the minimum positive intensity for each protein. All protein intensities were normalized to total protein content in each sputum sample, measured with the bicinchoninic acid (BCA) assay (Thermo Fisher Scientific, Waltham, MA, USA).

#### 4.7.3. Univariate and Multivariate Analyses

Clinical and demographic variables were compared between COPD and pre-COPD groups using the Mann–Whitney U test for continuous variables and the χ^2^ test for categorical variables.

For each omics dataset, differences in feature abundance between COPD and pre-COPD groups were assessed using two-sample statistical tests, with *p*-values adjusted for multiple comparisons using the Benjamini–Hochberg FDR [36]. Features with FDR < 0.05 were considered statistically significant. Global data structure was examined with principal component analysis (PCA). Supervised separation was tested by partial least squares–discriminant analysis (PLS-DA) [37].

#### 4.7.4. Pathway Enrichment Analysis

For metabolomic and lipidomic datasets, pathway enrichment was performed in MetaboAnalyst 6.0 using Kyoto Encyclopedia of Genes and Genomes (KEGG) pathway mapping. Proteomic data were analyzed with ProteinScape’s integrated functional annotation tools, applying KEGG pathway mapping to proteins detected in at least four samples. Separately, enrichment analysis of differentially expressed genes was performed using the Bioplanet pathway database from EnrichR database, using a hypergeometric test with Benjamini–Hochberg correction [34]. Statistical significance was defined as an adjusted *p*-value ≤ 0.05 and *p*-value < 0.05 for protein enrichment analysis.

#### 4.7.5. Interaction Network Analysis

Interaction network analysis was performed for differentially expressed proteins and proteins associated with COPD extracted from DisGeNet database (Chronic Obstructive Airway Disease, C0024117) [38]. Interactions for these two combined lists of genes were obtained from human interactome through String app for Cytoscape software (version 3.10.2) [39,40]. For more precise visualization, we extracted only connected nodes and interactions between differentially expressed protein-related genes and COPD-related genes. Tissue expression confidence was obtained through StringApp and Tissues 2.0 database.

#### 4.7.6. Software and Statistical Thresholds

All statistical analyses were performed using MetaboAnalyst (version 6.0), ProteinScape (version 4.2), JASP (version 0.19.0.0), and R (version 4.5.1). Unless specified otherwise, statistical significance was defined as *p* < 0.05. Exact *p*-values are provided in the Results and Supplementary Tables.

## Figures and Tables

**Table 1:** Demographic and clinical characteristics.

Basic Demographics and General Status	COPD (*n* = 28)	pre-COPD (*n* = 16)	*p*-Value
Age, yr	65.96 ± 7.53	64.13 ± 11.20	0.798
Female sex, *n* (%)	11 (39.3)	8 (50.0)	0.490
BMI, kg m^−2^	26.63 ± 4.84	26.53 ± 2.94	0.932
Current smoker, *n* (%)	24 (85.7)	10 (62.5)	0.077
Pack-years	50.30 ± 27.57	34.53 ± 10.71	0.035
Duration of COPD, yr	3.80 ± 4.92	-	-
mMRC Dyspnea Scale	1.64 ± 0.87	0.31 ± 0.60	<0.001
CAT score	13.79 ± 5.11	8.69 ± 5.99	0.007
Hyperlipidaemia, *n* (%)	7 (25.0)	2 (12.5)	0.323
Lipid lowering medication, *n* (%)	10 (35.7)	4 (25.0)	0.463
Vitamin D supplementation, *n* (%)	6 (21.4)	5 (31.2)	0.512
Omega-3 supplementation, *n* (%)	3 (10.7)	1 (6.3)	0.596

Values for continuous variables are presented as means ± standard deviations; categorical variables are presented as *n* (%), where percentages represent the proportion of participants within each study group. *Abbreviations:* BMI, Body Mass Index; CAT, COPD Assessment Test; COPD, Chronic Obstructive Pulmonary Disease; mMRC, modified Medical Research Council; SD, standard deviation.

**Table 2:** Pulmonary function tests.

	COPD (*n* = 28)		pre-COPD (*n* = 16)		*p*-Value
	*M*	*SD*	*M*	*SD*	
Spirometry					
FEV_1_ (L)	1.71	0.60	2.84	0.95	<0.001
FEV_1_ (z-score)	−2.04	1.01	0.04	0.99	<0.001
FVC (L)	3.54	1.04	3.85	1.17	0.373
FVC (z-score)	0.43	1.03	0.35	0.94	0.951
FEV_1_/FVC (%)	48.73	10.12	73.26	4.53	<0.001
FEV_1_/FVC (z-score)	−3.28	0.84	−0.56	0.53	<0.001
Body plethysmography					
TLC (L)	6.50	1.15	6.01	1.62	0.130
TLC (z-score)	1.57	1.07	0.39	1.05	0.003
RV (L)	2.77	0.51	2.14	0.49	<0.001
RV (z-score)	1.74	1.42	−0.03	0.99	<0.001
Diffusing					
DLCO (z-score)	−1.95	0.82	−1.48	1.02	0.227

Variables are presented as mean (M) and standard deviation (SD) values. *Abbreviations:* FEV_1_, Forced Expiratory Volume in 1 s; FVC, Forced Vital Capacity; TLC, Total Lung Capacity; RV, Residual Volume; DLCO, Diffusing Capacity of the Lung for Carbon Monoxide.

## Data Availability

The mass spectrometry data have been deposited to the ProteomeXchange Consortium via the PRIDE [41] partner repository with the dataset identifier PXD067791.

## Supplementary Materials

The following supporting information can be downloaded at: https://www.mdpi.com/article/10.3390/ijms27052271/s1.

## Abbreviations

The following abbreviations have been used in this manuscript:

ABE	GOLD 2022 ABE categories
ATS	American Thoracic Society
BCA	Bicinchoninic Acid
BMI	Body Mass Index
CAT	COPD Assessment Test
COPD	Chronic Obstructive Pulmonary Disease
CRP	C-reactive Protein
DLCO	Diffusing Capacity of the Lung for Carbon Monoxide
ECM	Extracellular Matrix
ERS	European Respiratory Society
ESI	Electrospray Ionization
FEV_1_	Forced Expiratory Volume in 1 Second
FDR	False Discovery Rate
FTICR	Fourier-Transform Ion Cyclotron Resonance
FVC	Forced Vital Capacity
GOLD	Global Initiative for Chronic Obstructive Lung Disease
HRCT	High-Resolution Computed Tomography
JAK	Janus Kinase
KEGG	Kyoto Encyclopedia of Genes and Genomes
LC-MS/MS	Liquid Chromatography–Tandem Mass Spectrometry
LLN	Lower Limit of Normal
LoD	Limit of Detection
mMRC	Modified Medical Research Council
NO	Nitric Oxide
NT-proBNP	N-terminal pro-B-type Natriuretic Peptide
PCA	Principal Component Analysis
PLS-DA	Partial Least Squares–Discriminant Analysis
PRIDE	PRoteomics IDEntification Database
QC	Quality Control
Rho	Ras homologous small GTPase
RV	Residual Volume
S1P	Sphingosine-1-Phosphate
SD	Standard Deviation
STAT	Signal Transducer and Activator of Transcription
TLC	Total Lung Capacity
UHPLC	Ultra-High-Performance Liquid Chromatography
