# Supplementary material for: Induced Sputum Multi-Omics Reveals Airway Signatures of COPD in Smokers: A Pilot Study
Source: Int J Mol Sci. 2026 Feb 28;27(5):2271. doi: 10.3390/ijms27052271 (PMC12984585; doi:10.3390/ijms27052271)
Supplement: Supplementary file 1 [file ijms-27-02271-s001.zip › ijms-4120996-supplementary.pdf]

M1. Lipidomic Analysis

Sample preparation

Induced sputum samples (30 μL) were combined with 225 μL of methanol (hyper LC-MS grade, Merck, Darmstadt, Germany) and vortexed for 10 seconds. Next, 750 μL of methyl tert-butyl ether (Merck) was added, and the mixture was incubated on a shaker at room temperature for 1 hour. Following incubation, 190 μL of water was added, and the samples were vortexed for 10 seconds and centrifuged at 1,000 × g for 10 minutes at room temperature. The upper organic phase (~500 μL) was collected into a new tube and dried overnight using a vacuum centrifuge (SpeedVac). Dried extracts were stored at –20°C until mass spectrometry analysis.

Mass spectrometry acquisition

Mass spectra were acquired in both positive-ion and negative-ion electrospray ionization (ESI) modes using a 12 T SolariX FTICR mass spectrometer (Bruker Daltonics). The ion source was operated at 4,500 V, with nitrogen drying gas at 4 L/min. The end-plate offset was set to –200 V. Collision energy was −5 V, with a time-of-flight (TOF) of 1.2 ms. Data were acquired in CASI mode (700–1,000 m/z window) at a frequency of ~0.3 Hz, with a mass accuracy better than 3 ppm.

Data processing

Calibration was performed before acquisition using sodium trifluoroacetate (NaTFA) in both ionization modes. Extracted ion chromatograms (0.01 Da width) were used for peak integration. Data was processed using the T-Rex 2D algorithm (MetaboScape 5.0, Bruker Daltonics). Lipid annotation was performed using the Lipid Maps 2018 and Bruker Lipid MetaboScape databases. False-positive peaks were removed prior to statistical analysis.

M2. Metabolomic Analysis

Sample preparation

Induced sputum samples were lysed with 145 μL of ice-cold methanol (–20°C, hyper LC-MS grade, Merck) and incubated at -20°C for 2 hours, with vortexing every 20 minutes. Samples were centrifuged at 16,000 × g for 20 minutes at 10°C. A 150 μL aliquot of the supernatant was lyophilized in a vacuum concentrator (Eppendorf SE, Hamburg, Germany). Dried extracts were reconstituted in 2% methanol and centrifuged at 14,000 × g for 10 minutes at 4°C to remove particulates.

Mass spectrometry acquisition

Spectra were collected using the same 12 T SolariX FTICR MS platform in both positive-ion and negative-ion ESI modes. Acquisition parameters matched those used in lipidomics: ion source voltage 4,500 V, nitrogen drying gas 4 L/min, end-plate offset −200 V, collision voltage −5 V, TOF 1.2 ms, CASI mode 700–1,000 m/z, frequency ~0.3 Hz, and mass accuracy <3 ppm.

Data processing

Calibration was performed with NaTFA in both ionization modes. Extracted ion chromatograms (0.01 Da width) were integrated. Annotation used the HMDB and Lipid Maps databases. Quality control (QC) samples procedures included pooled quality control samples and mass calibration before each run. Data were processed in MetaboScape 5.0 with false-positive removal.

M3. Proteomic Analysis

Sample preparation

Fifty micrograms of induced sputum was transferred into low-binding Eppendorf tubes (Eppendorf SE). Samples were incubated at 96°C for 60 minutes, then sonicated for 90 minutes in a water bath. Protein precipitation was performed using ice-cold (−20°C) acetonitrile (ACN, Merck) at a 1:4 ratio, followed by incubation at −20°C for 120 minutes. Samples were centrifuged at 18,000 × g for 30 minutes at −9°C, and the supernatant was removed. Protein pellets were dissolved in 40 mM ammonium bicarbonate (Merck). Reduction was performed with 500 mM DTT (final concentration 20 mM), followed by alkylation with 1 M iodoacetamide (IAA, final concentration 40 mM). Proteins were digested in-solution with Trypsin Gold (Promega, Madison, WI, USA) for 16 hours at 37°C.

Mass spectrometry acquisition

Peptides were separated on a nano-UHPLC system (nanoElute, Bruker Daltonics) with a pre-column (300 µm × 5 mm, C18 PepMap 100, 5 µm, 100 Å, Thermo Fisher Scientific) and an analytical column (75 µm × 100 mm, C18, 1.9 µm, Bruker Daltonics) using a gradient of 2–55% solvent B over 20 minutes at a flow rate of 300 nL/min. MS analysis was performed on a Compact ESI-QTOF mass spectrometer (Bruker Daltonics) equipped with a CaptiveSpray ion source. Spectra were calibrated using lock mass.

Data processing

Raw data were processed in DataAnalysis (Bruker Daltonics) and searched in ProteinScape (Bruker Daltonics) using the Mascot search engine against the SwissProt *Homo sapiens* database, with a peptide-level FDR of 1%.

Table S1. Summary of the top 5 metabolites (univariate t-test, COPD vs. pre-COPD).

	t.stat	p.value	-log10(p)	FDR	COPD	non- COPD
**PC 15:0/22:6**	-2.7635	0.0084538	2.0729	0.70371	↓	↑
**Glutathione**	2.398	0.021007	1.6776	0.70371	↑	↓
**N-(13Z-docosanoyl)-ethanolamine**	-2.2205	0.031836	1.4971	0.70371	↓	↑
**Isobutyryl-L-carnitine**	2.1652	0.036099	1.4425	0.70371	↑	↓
**Creatine**	2.1376	0.038411	1.4155	0.70371	↑	↓

Table S1. Top 5 metabolites identified by univariate t-test comparing COPD and pre-COPD groups. The table shows t-statistics, nominal p-values, negative log10-transformed p-values, and false discovery rate (FDR) values.

Table S2. Top 10 lipids differentiating COPD and pre-COPD subjects (univariate t-test).

	t.stat	p.value	-log10(p)	FDR	COPD	non-COPD

**8-hydroxy-5,6-Octadienoic acid**	3.4716	0.001	2.9167	0.62383	↑	↓
**(8E)-10-oxodec-8-enoic acid**	3.1295	0.003	2.4974	0.62383	↑	↓
**2'-O-(alpha-D-Manp)-(1-heptadecanoyl-2-octadecanoyl-sn-glycero-3-phospho-1'-myo-inositol)**	3.0076	0.004	2.3532	0.62383	↑	↓
**2-(9R-(5Z,9Z-tetracosadienoyloxy)-3-methyl-2Z-decenoyloxy)-ethanesulfonic acid**	2.6049	0.013	1.8977	0.62383	↑	↓
**PG 22:3**	2.5548	0.014	1.8435	0.62383	↑	↓
**heptadecasphinganine**	2.4851	0.017	1.769	0.62383	↑	↓
**N-(hexadecanoyl)-sphing-4-enine**	2.4669	0.018	1.7499	0.62383	↑	↓
**2E,4E,8E,10E-Dodecatetraenedioic acid**	2.4205	0.02	1.7011	0.62383	↑	↓
**11-amino-undecanoic acid**	2.3999	0.021	1.6796	0.62383	↑	↓
**9-oxo-heptadecanoic acid**	-2.3832	0.022	1.6623	0.62383	↓	↑

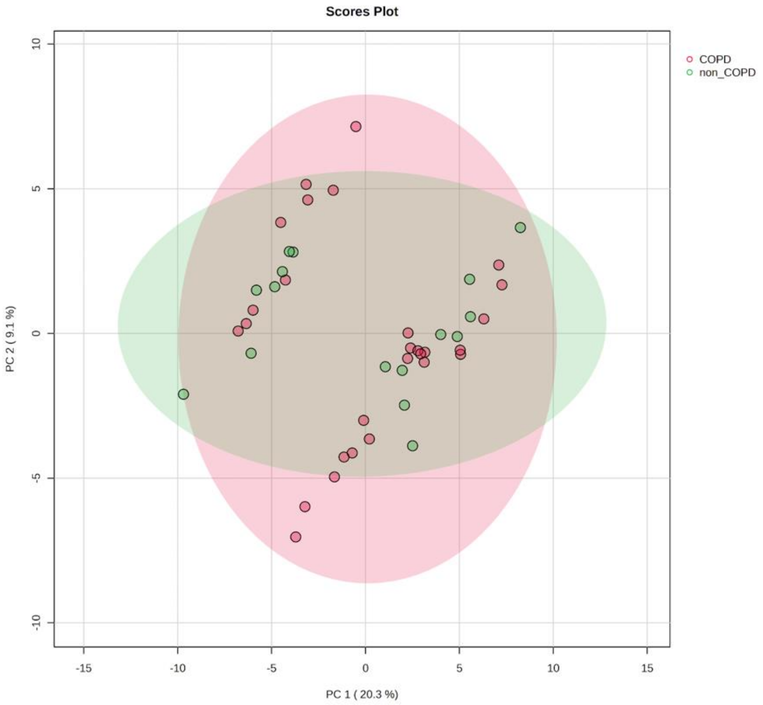

Figure S1. Principal component analysis (PCA) of metabolomic profiles in COPD and pre-COPD subjects. Each point represents a participant; color indicates clinical group. PC1 and PC2 explain 20.3% and 9.1% of variance, respectively.

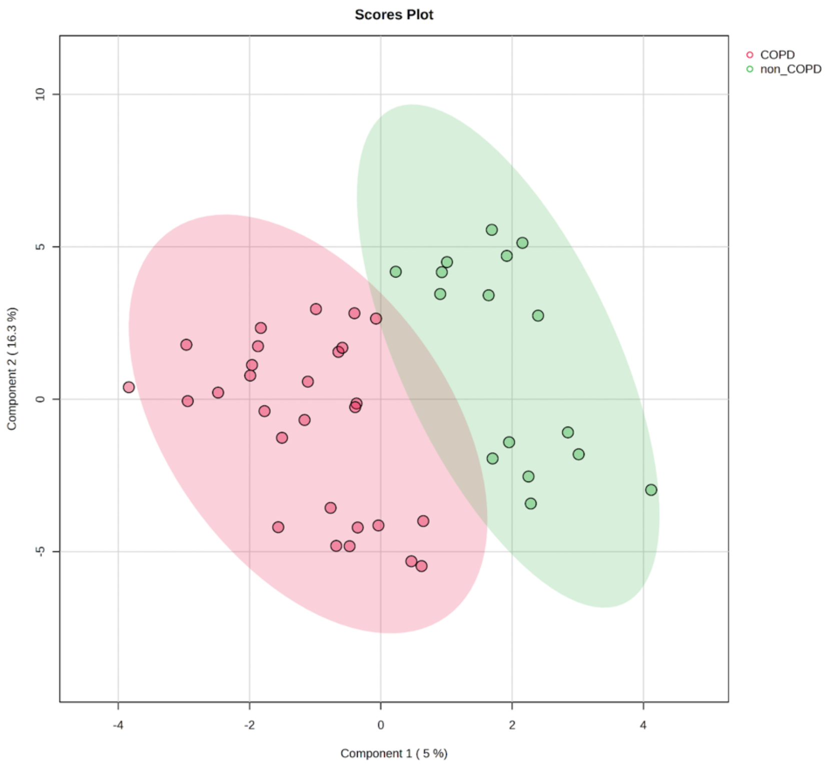

Figure S2. Partial least squares discriminant analysis (PLS-DA) score plot of metabolomic profiles, showing separation of COPD and pre-COPD groups. Components 1 and 2 explain 5% and 16.3% of variance, respectively.

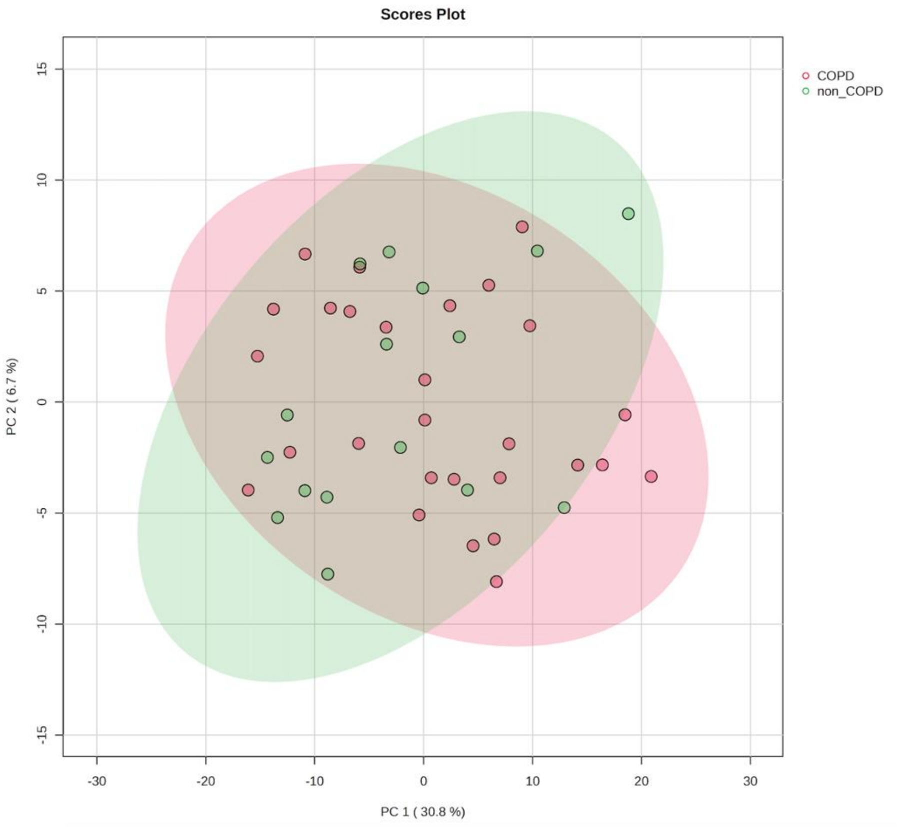

Figure S3. Principal component analysis (PCA) of lipidomic profiles in COPD and pre-COPD subjects. Each point represents a participant; color indicates clinical group. PC1 and PC2 explain 20.3% and 9.1% of variance, respectively.

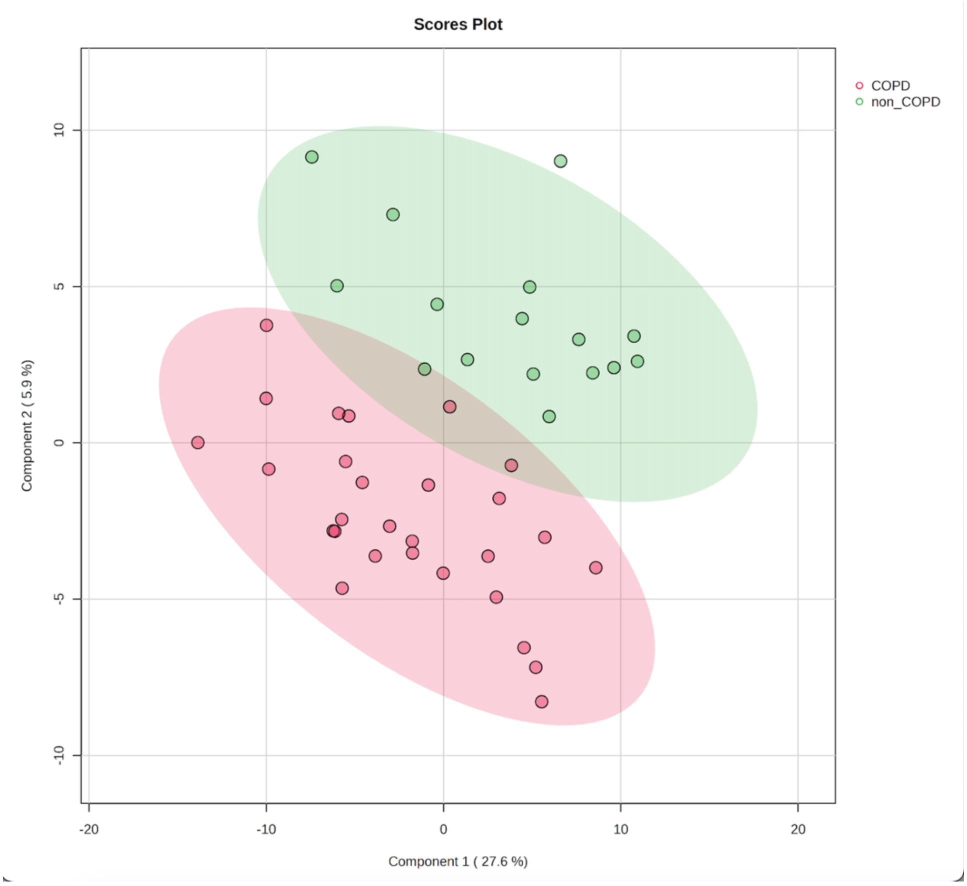

Figure S4. Partial least squares discriminant analysis (PLS-DA) score plot of lipidomic profiles, showing partial separation of COPD and pre-COPD groups.
